# Radiotranscriptomics signature‐based predictive nomograms for radiotherapy response in patients with nonsmall cell lung cancer: Combination and association of CT features and serum miRNAs levels

**DOI:** 10.1002/cam4.3115

**Published:** 2020-05-27

**Authors:** Liyuan Fan, Qiang Cao, Xiuping Ding, Dongni Gao, Qiwei Yang, Baosheng Li

**Affiliations:** ^1^ Cheeloo College of Medicine Shandong University Jinan Shandong China; ^2^ Department of Radiation Oncology Shandong First Medical University and Shandong Academy of Medical Sciences Shandong Cancer Hospital and Institute Huaiyin Region Jinan Shandong China; ^3^ School of Computer Science and Engineering Southeast University Nanjing Jiangsu China

**Keywords:** CT texture features, miRNAs, nomogram, nonsmall cell lung cancer, radiotherapy response

## Abstract

**Purpose:**

We aimed to establish radiotranscriptomics signatures based on serum miRNA levels and computed tomography (CT) texture features and develop nomogram models for predicting radiotherapy response in patients with nonsmall cell lung cancer (NSCLC).

**Methods:**

We first used established radioresistant NSCLC cell lines for miRNA selection. At the same time, patients (103 for training set and 71 for validation set) with NSCLC were enrolled. Their pretreatment contrast‐enhanced CT texture features were extracted and their serum miRNA levels were obtained. Then, radiotranscriptomics feature selection was implemented with the least absolute shrinkage and selection operator (LASSO), and signatures were generated by logistic or Cox regression for objective response rate (ORR), overall survival (OS), and progression‐free survival (PFS). Afterward, radiotranscriptomics signature‐based nomograms were constructed and assessed for clinical use.

**Results:**

Four miRNAs and 22 reproducible contrast‐enhanced CT features were used for radiotranscriptomics feature selection and we generated ORR‐, OS‐, and PFS‐ related radiotranscriptomics signatures. In patients with NSCLC who received radiotherapy, the radiotranscriptomics signatures were independently associated with ORR, OS, and PFS in both the training (OR: 2.94, *P* < .001; HR: 2.90, *P* < .001; HR: 3.58, *P* = .001) and validation set (OR: 2.94, *P* = .026; HR: 2.14, *P* = .004; HR: 2.64, *P* = .016). We also obtained a satisfactory nomogram for ORR. The C‐index values for the ORR nomogram were 0.86 [95% confidence interval (CI), 0.75 to 0.92] in the training set and 0.81 (95% CI, 0.69 to 0.89) in the validation set. The calibration‐in‐the‐large and calibration slope performed well. Decision curve analysis indicated a satisfactory net benefit.

**Conclusions:**

The radiotranscriptomics signature could be an independent biomarker for evaluating radiotherapeutic responses in patients with NSCLC. The radiotranscriptomics signature‐based nomogram could be used to predict patients’ ORR, which would represent progress in individualized medicine.

## Graphical abstract

Serum miRNAs expression levels and CT texture features could be combined to develop radiotranscriptomics signature for independently predicting radiotherapy response. Radiotranscriptomics signature‐based nomogram was constructed for objective response rates. The statistical significance between serum miRNAs levels and CT texture features was preliminarily explored.

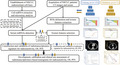

## 1 INTRODUCTION

Lung cancer has a high incidence worldwide, second only to prostate cancer in males and breast cancer in females.^1^ It is also the most lethal cancer type and causes one‐quarter of all cancer deaths worldwide.^1^ The 5‐year overall survival (OS) of lung cancer patients is less than 20% and that of patients with distant metastasis is only approximately 5%.^2^ More than 50% of lung cancer patients receive radiotherapy for both thoracic disease and extra thoracic metastatic sites.^3^ In contrast to small cell lung cancer (SCLC), patients with nonsmall cell lung cancer (NSCLC) exhibit wide individual heterogeneity in radiotherapeutic effects. Therefore, it is of significant benefit to predict the prognosis of patients with NSCLC for optimal treatment.

Many efforts have been made to achieve this aim, and the most attention has been paid to molecular and image features. With the development of next‐generation sequencing, many genomic biomarkers, such as DNA polymorphisms and RNA expression levels, have emerged in recent years in the field of “genomics”.^4^ These biomarkers have been used for both diagnosis and prognostic prediction in many cancer types, including NSCLC.^5^ In addition, with the maturation of image‐processing technology, including computed tomography (CT), magnetic resonance Imaging (MRI), PET‐CT, etc, “radiomics” has also emerged.^6^ It indicates that image features have a potential to influence clinical decision‐making as well as therapy planning for radiation oncology.^6^, ^7^ Of interest is that these two “‐mics” could work together for this topic and may be related to each other. As a result, “radiogenomics” was created, which combines “radiomics” and “genomics” for both radiotherapy response modeling and prognostic implications in patients with NSCLC and other cancer types.^8^, ^9^, ^10^ Radiotranscriptomics,^11^ a branch of radiogenomics, which combines “radiomics” and “transcriptomics” (like mRNA, miRNA, lncRNA expression levels), could also be used as tumor biomarkers. However, it has not yet been fully explored, especially regarding the combination and association between miRNAs and CT image features in NSCLC.

In this study, we aimed to combine serum miRNA levels and contrast‐enhanced CT texture features to construct radiotranscriptomics signatures and related nomograms for predicting ORR, OS, and PFS in patients with NSCLC. We also preliminarily explored the relationship between serum miRNA levels and CT texture features.

## 2 MATERIAL AND METHODS

### 2.1 Study design and patient collection

**Figure 1:**
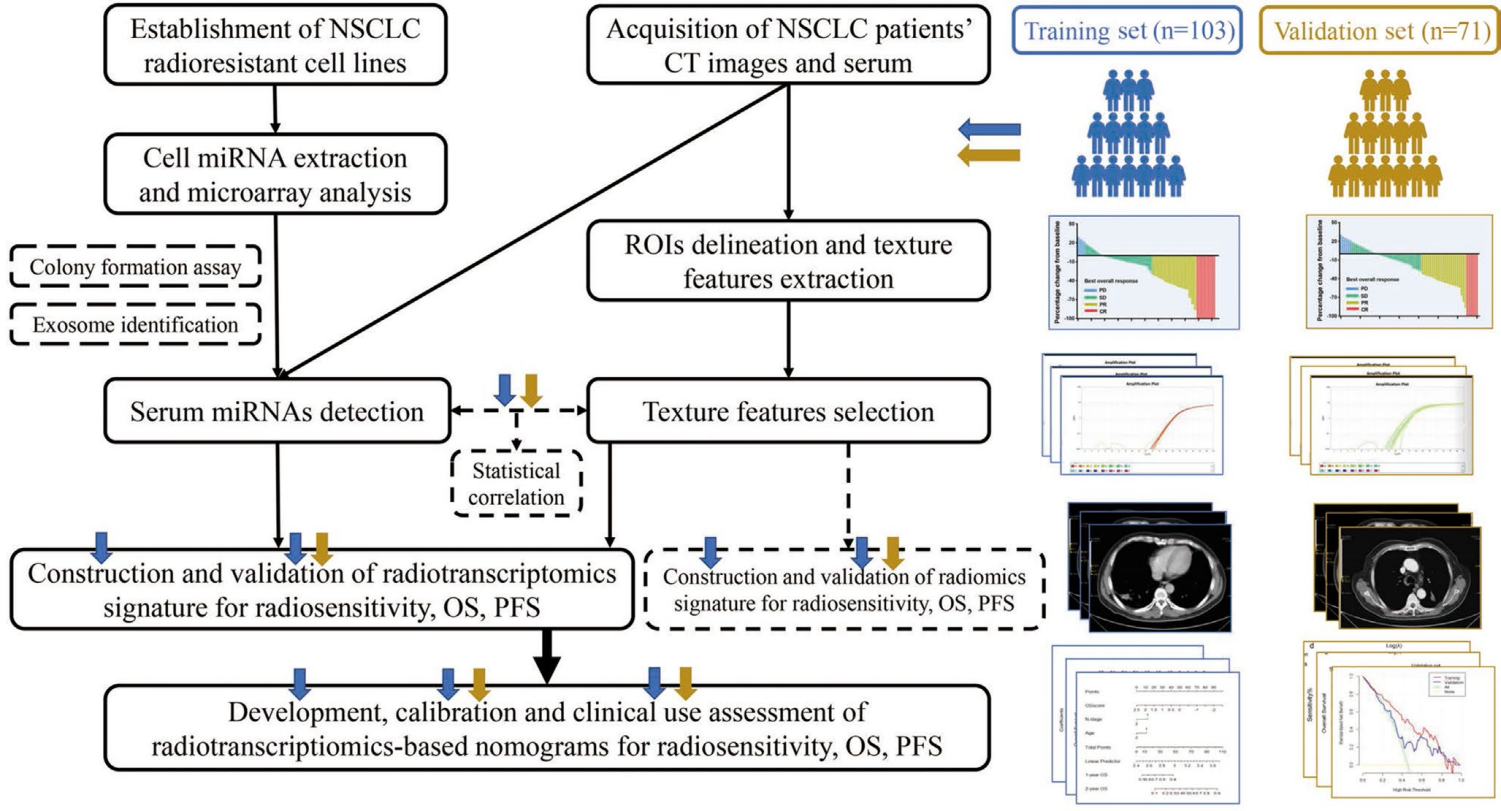
Flow diagram in this study. CT, computerized tomography; NSCLC, nonsmall cell lung cancer; PFS, progression‐free survival; ROI, region of interest; OS, overall survival

We performed a prospective study to identify potential radiotranscriptomics biomarkers to predict the radiotherapeutic response of patients with NSCLC (Figure 1). With approval of the institutional ethical committee, we enrolled 103 patients with NSCLC from November 2014 to October 2015 for the training set and 71 patients with NSCLC from November 2015 to May 2016 for the validation set. The inclusion criteria were as follows: (a) patients with NSCLC who received radiotherapy either combined or not combined with chemotherapy; and (b) availability of pretreatment serum and contrast‐enhanced CT scans within 1 month as well as follow‐up information for 40 months. The exclusion criteria were patients who received thoracic surgery before or after radiotherapy. All patients’ basic characteristics are summarized in Table 1. The radiotherapy response of the primary sites was evaluated according to RECIST 1.1^12^ (Additional file 1: Figure S1) and recorded as the objective response rate (ORR). For survival assessment, both overall survival (OS) and progression‐free survival (PFS) were used as endpoints.

**Table 1:** Basic characteristics of patients in the training set and validation set

Characteristics	Training set (n = 103)	Validation set (n = 71)	*P* ^a^
Age(yr)			
≤60	48 (46.60%)	34 (47.89%)	0.867
>60	55 (53.40%)	37 (52.11%)	
Sex			
Women	40 (38.83%)	29 (40.85%)	0.790
Men	63 (61.17%)	42 (59.15%)	
Pathology			
AC	51 (49.51%)	38 (53.52%)	0.603
SCC	52 (50.49%)	33 (46.48%)	
Differentiation			
Well and Moderate	46 (44.66%)	31 (43.66%)	0.896
Poor	57 (55.34%)	40 (56.34%)	
Stage			
I ~ II	18(17.48%)	13(18.31%)	0.990
III	57 (55.34%)	39(54.93%)	
IV	28 (27.18%)	19(26.76%)	
T stage			
T1 and T2	46 (44.66%)	33(46.48%)	0.748
T3 and T4	57 (55.34%)	37(53.52%)	
N stage			
N0 and N1	35 (33.98%)	24(33.80%)	0.981
N2 and N3	68 (66.02%)	47(66.20%)	
M stage			
M0	75 (72.82%)	52 (73.24%)	0.951
M1	28 (27.18%)	19 (26.76%)	
Chemotherapy			
N	45 (43.69%)	30 (42.25%)	0.851
P or NP	58 (56.31%)	41 (57.75%)	

Note

Abbreviations: AC, adenocarcinoma; N, Nonplatinum drugs; NP, both nonplatinum and platinum drugs; P, platinum drugs; SCC, squamous cell carcinoma.

^a^ Compared using *χ* ^2^ test.

### 2.2 MIRNA extraction, microarray analysis, and QRT‐PCR

We established radioresistant (RR) cell lines (A549‐R and PC9‐R) according to the method described in a previous study (accepted for publication elsewhere). Total RNA from cells and serum was extracted by using TRIzol Reagent (Ambion, USA). The RNA concentration and purity were measured by a Model 680 Microplate Reader (Bio‐Rad, USA), while the integrity was assessed by electrophoresis on a denaturing agarose gel.

To obtain the differentially expressed (DE) miRNA profile, a microarray was carried out (under consideration for publication elsewhere) at the Beijing Bioassay Laboratory of CapitalBio Corporation by the Human MiRNA Microarray, Release 21.0, 8 × 60K (Agilent Technologies) according to the manufacturer's protocol. To verify the microarray results and identify potential clinical biomarkers, we ranked DE miRNAs according to the fold changes, q‐value, and relative expression value. The top‐ranked miRNAs with the greatest fold changes were selected, including miR‐2861, miR‐4298, miR‐1290, miR‐92a‐1‐5p, and miR‐25‐5p. Quantitative reverse transcription polymerase chain reaction **(**qRT‐PCR) was performed by using SYBR Green Master Mix (GeneCopoeia, USA) on an LC480 system (Roche, Switzerland). The primers are listed in Additional file 10: Table S1. All samples were assayed three times independently with replicating each three times. The results were analyzed by the 2^−ΔΔCt^ method and normalized against internal controls (U6).

### 2.3 Cell culture and colony formation assays

Cells were seeded at a density of 1 × 10^6^ cells/60 mm culture dish with complete medium. Twenty‐four hours later, we transfected miR‐2861 and miR‐1290 mimics (GenePharma) into A549/PC9 cells with high levels of endogenous expression and miR‐92a‐1‐5p and miR‐25‐5p inhibitors (GenePharma) into A549/PC9 cells with high levels of endogenous expression. The survival fractions (SFs) were calculated as follows: SF = Number of Colonies Counted/Number of Cells Seeded× (Plating Efficiency/100). The survival curve was derived using the L‐Q model: SF = exp(−(*α* × *D* + *β* × (*D* ^2^))).

### 2.4 Exosome identification from cell‐conditioned medium (CCM)

The A549, PC9, A549‐R, and PC9‐R cell lines were cultured in serum‐free media for at least 3 days and used for exosome isolation at 24, 48, and 72 hours. To remove detached cells, CCM was harvested and centrifuged at 10 000 × g at 48°C for 30 minutes. The supernatant was recycled and then centrifuged in a Beckman Coulter Optima^TM^ L80XP Ultracentrifuge at 100 000 × *g*_avg_ at 4℃ for 120 minutes with a Type 90 Ti rotor (k‐factor: 48) to pellet exosomes.

### 2.5 CT image acquisition, segmentation, and texture feature extraction

Contrast‐enhanced CT images were acquired at a tube potential of 120 kV with a tube current of 190‐600 mA and reconstructed at a slice thickness of 1.5‐2.5 mm with a resolution of 0.98 × 0.98‐1.17 × 1.17 mm^2^. The primary tumor site was contoured using both the soft tissue and lung windows, and two other radiation oncologists were invited to individually verify all contours. If a patient presented with more than one primary tumor site, the union of all sites would be analyzed.

LIFEx^13^ software was used for CT texture feature extraction from delineated three‐dimensional (3D) ROIs. Several categories of texture features were used to analyze the original images, including histogram, shape, GLCM matrix, GLRLM matrix, NGLDM matrix, and GLZLM matrix (Additional file 11: Table S2). For the GLCM matrix, three different distances of neighbors were used for analysis. Their detailed description was previously reported.^14^

### 2.6 Radiomics or radiotranscriptomics signature construction and validation

The intraclass correlation coefficient (ICC)^15^ was calculated to assess feature reproducibility in repeated delineation (ICC < 0.40, poor; 0.40 ≤ ICC <0.60, moderate; 0.60 ≤ ICC <0.80, good; ICC ≥ 0.80, excellent). Pairwise correlations among the above‐selected features (ICC ≥ 0.80) were also considered. After obtaining the optimal CT texture features, the least absolute shrinkage and selection operator (LASSO) model was used for feature selection. Then, multivariable logistic regression analysis was conducted to develop ORR signatures while Cox regression was to develop OS and PFS signatures, for both radiomics and radiotranscriptomics features. The radiotranscriptomics signatures along with basic clinical information were tested by multivariable analysis in both the training set and validation set.

### 2.7 Nomogram development, calibration, and assessment

In screening factors to include in the nomogram, the following aspects were considered. First, we used a multivariable logistic regression model to identify the independent predictors for radiosensitivity and a Cox regression model for OS or PFS. Second, we referred to previous research on nomogram construction. Moreover, we also took our actual patients’ information and conditions as well as clinical practice into account. The C‐index (discrimination), calibration‐in‐the‐large, and calibration slope (calibration) obtained by plotting the actual probability and the nomogram‐predicted probability were used to measure the performance of the nomograms in both the training and validation sets. Finally, decision curve analysis (DCA) was conducted to calculate the net benefits at different threshold probabilities for clinical use assessment.

### 2.8 Statistical analysis

The areas under the receiver operating characteristic (ROC) curves (AUCs) were used to assess the efficacy of predicting ORR, while Kaplan‐Meier (K‐M) curves were used to assess the ability to discriminate OS or PFS. GraphPad Prism 7.0 was used to perform statistical tests (independent *t* tests and *χ* ^2^ test under the conditions described below) and image processing. R software (version 3.5.1.) was used for signature feature selection, nomogram development, and assessment.

## 3 RESULTS

### 3.1 Functional demonstration and origin exploration of mi RNAs related to radiosensitivity in NSCLC cells

**Figure 2:**
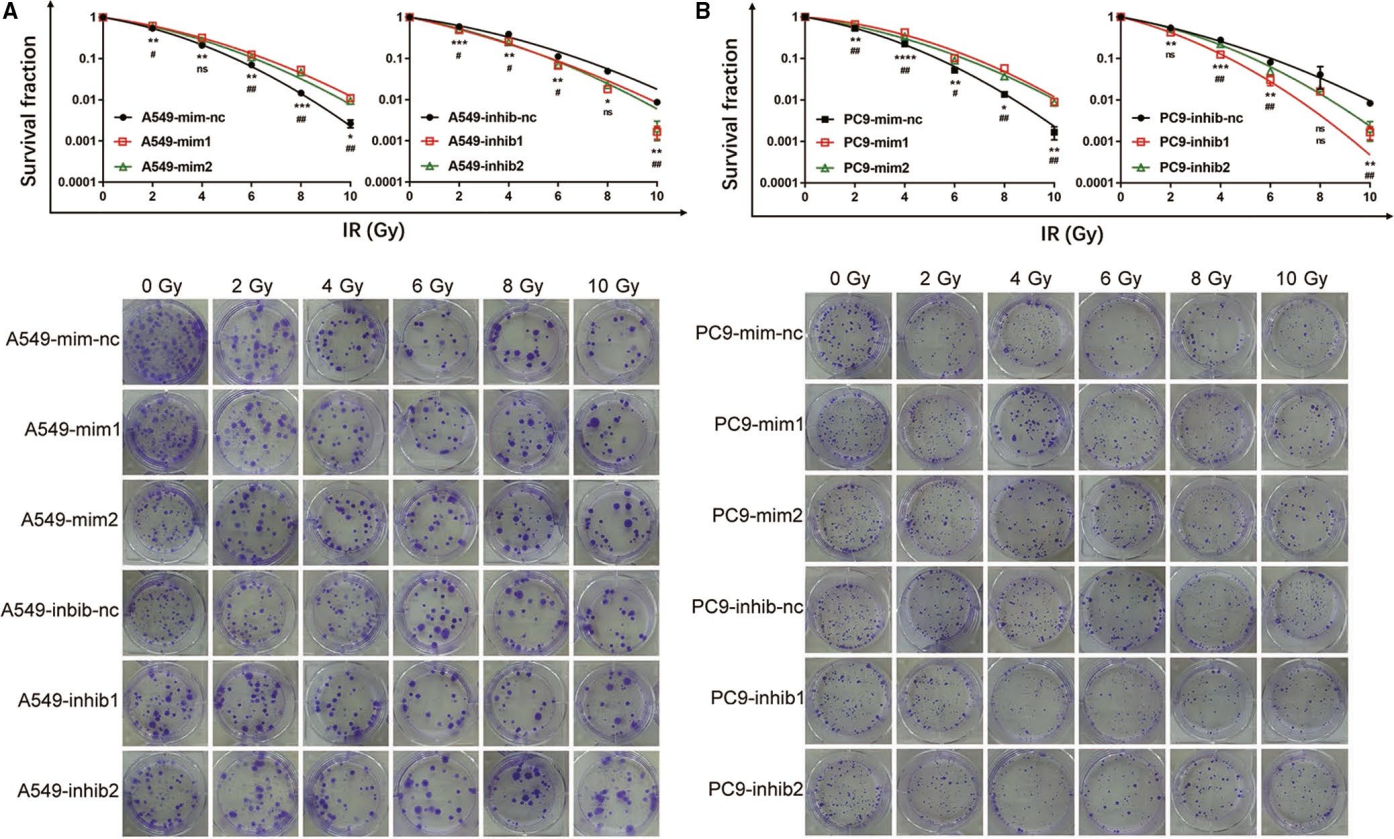
MiRNAs increase or decrease NSCLC cells’ radiosensitivity in vitro. n = 3 per group. mim1 = miR‐1290 mimics, mim2 = miR‐2861 mimics, inhib1 = miR‐25‐5p inhibitor, inhib2 = miR‐92a‐1‐5p inhibitor

The qRT‐PCR results show that four miRNAs (miR‐2861, miR‐1290, miR‐92a‐1‐5p, and miR‐25‐5p) are consistent with the microarray results (Additional file 12: Table S3). The colony formation assay for the functional demonstration demonstrates that miR‐2861 and miR‐1290 decreased radiosensitivity, while miR‐25‐5p and miR‐92a‐1‐5p increased radiosensitivity in NSCLC cell lines (Figure 2). The detailed data are presented in Additional file 13: Table S4.

Several previous studies have shown the potential of serum miRNAs as tumor biomarkers, and we preliminarily explored the origin of serum miRNAs in vitro. After a literature review, we hypothesized that the serum miRNAs came from exosomes that were secreted by tumor cells and wondered if there was a quantity difference between radioresistant and parent cell lines. Therefore, we collected CCM to isolate exosomes and detected miRNAs by qRT‐PCR. The results show that the levels of miR‐1290 and miR‐2861 in A549 and PC9 cells are lower than those in A549‐R and PC9‐R cells, while the levels of miR‐25‐5p and miR‐92a‐1‐5p in A549 and PC9 cells are higher than those in A549‐R and PC9‐R cells (Additional file 2: Figure S2). Moreover, the levels of these four miRNAs increase in a time‐dependent manner.

### 3.2 Construction and validation of radiotranscriptomics signature

**Figure 3:**
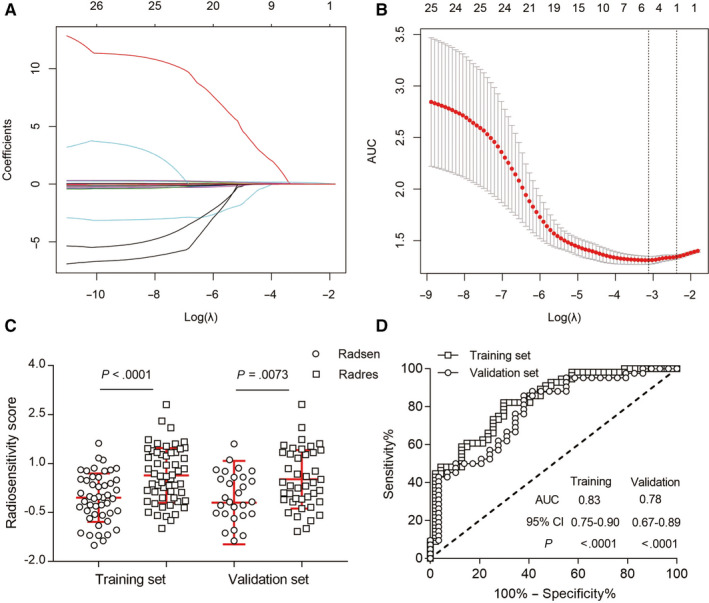
Radiotranscriptomics features selection and validation for objective response rate (ORR). A, The least absolute shrinkage and selection operator (LASSO) coefficient profiles of the 22 most stable texture features and 4 miRNAs expression levels. B, Tuning parameter (*λ*) selection in the LASSO model used 10‐fold cross‐validation via minimum criteria. A *λ* value of 0.044 was chosen according to 10‐fold cross‐validation. C, The radiotranscriptomics scores were calculated and classified into radiosensitive and radioresistant groups in both training and validation sets. Radsen = radiosensitive patients, Radres = radioresistant patients. D, Receiver operating characteristic (ROC) curve analysis of radiotranscriptomics scores in both training and validation sets. AUC, area under curve; CI, confidence interval

As listed in Additional file 11: Table S2, we extracted 52 CT texture features by using LifeX. After reproducible selection, 22 image features, including 3 histogram features, 2 shape features, 8 GLCM matrix features, 3 GLRLM matrix features, 1 NGLDM matrix feature, and 5 GLZLM matrix features, remained for further analysis (Additional file 3: Figure S3). Along with the miRNA features mentioned above, 4 radiotranscriptomics features with nonzero coefficients by LASSO regression (Figure 3A,B) in the training set were related to the ORR of patients with NSCLC who received radiotherapy. The radiotranscriptomics signature constructed by the logistic regression model is represented by the following the formula: ORR Score = 0.9442 + 0.01905 × GLCM_Correlation − 0.00002623 × GLCM_Entropy + 0.2027 × miR‐1290 + 0.08879 × miR‐2861. After calculating the ORR score of each patient in both the training and validation sets, we found that the ORR score was significantly lower in the radiosensitive group than in the radioresistant group (Figure 3C). ROC curves were also calculated, and the AUCs in the training and validation set were 0.83 [95% confidence interval (CI), 0.75 to 0.90] and 0.78 (95% CI, 0.67 to 0.89), respectively (Figure 3D).

**Figure 4:**
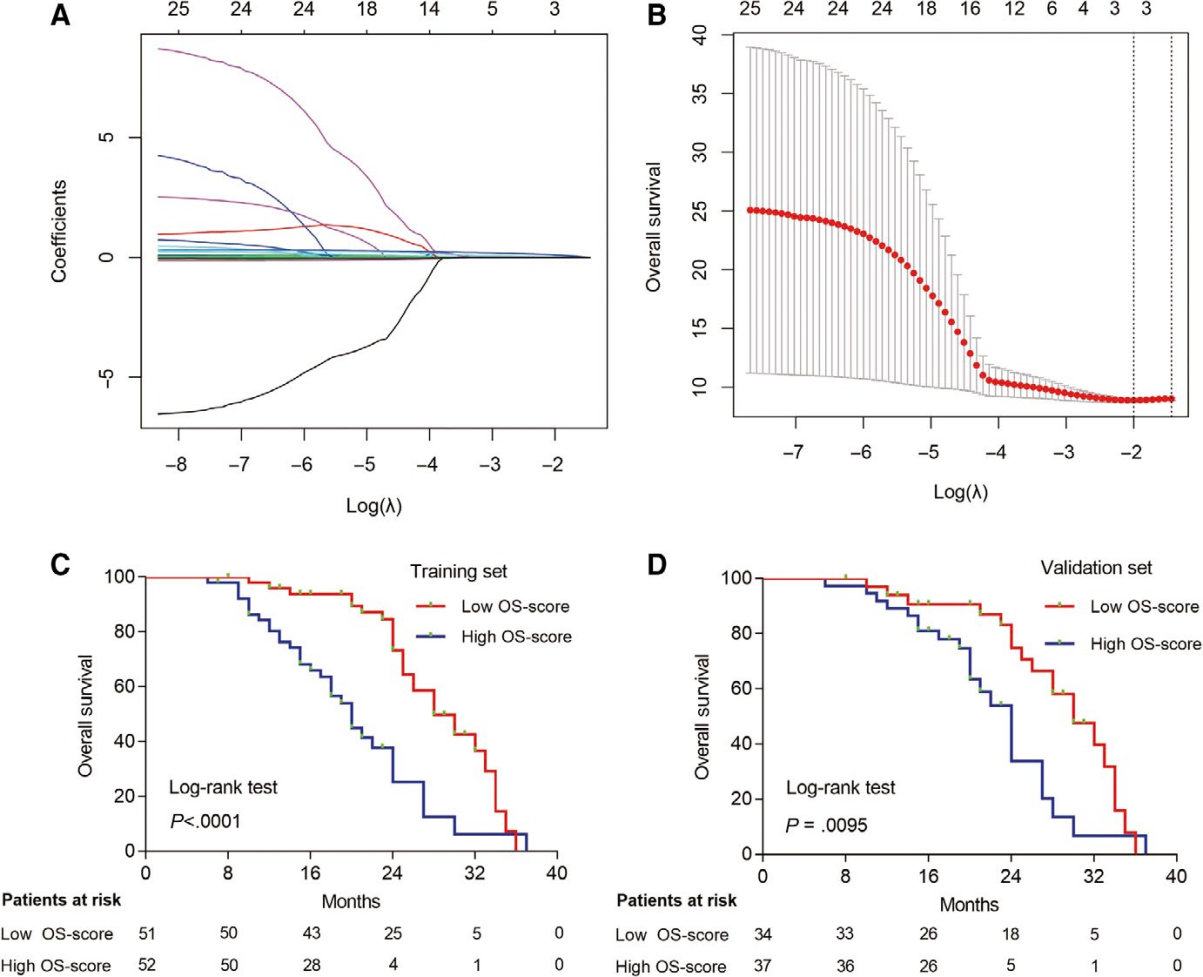
Radiotranscriptomics features selection and validation for overall survival (OS). A, The LASSO coefficient profiles of the 22 most stable texture features and 4 miRNAs expression levels. B, Tuning parameter (*λ*) selection in the LASSO model used 10‐fold cross‐validation via minimum criteria. A *λ* value of 0.135 was chosen according to 10‐fold cross‐validation. C,D, Kaplan‐Meier curve analysis of OS based on radiotranscriptomics score in both training and validation sets. The green dots indicated censored observations

Moreover, the OS‐ and PFS‐related radiotranscriptomics features were also selected by LASSO in the training set (Figure 4A,B, Additional file 4: Figure S4A,B), and the following signatures were generated by the Cox regression model as follows: OS Score = 0.026080 × Histogram_Energy + 0.219230 × miR.1290 + 0.079503 × miR.2861; PFS Score = −0.0076494 × GLCM_Contrast −0.0469175 × GLCM_Contrast.1 + 0.0003379 × GLZLM_LZLGE −0.0886312 × miR.25.5p. We chose the median scores as the cut‐off values, and K‐M curves were generated for both the training and validation sets. We found that the patients who had a lower OS score or PFS score had a longer OS (Figure 4C,D) or PFS (Additional file 4: Figure S4C,D) than patients who had higher OS scores. The predictive power of ORR‐, OS‐, and PFS‐related radiotranscriptomics signatures were superior to those of the radiomics signature (Additional files 5 and 6: Figures S5 and S6). It indicated that it is rational to combine transcriptomic s and radiomics features to predict radiotherapy response in patients with NSCLC. Multivariate analysis showed that these radiotranscriptomics signatures were independent factors for predicting patients’ ORR, OS, and PFS in both the training set (Additional file 14: Table S5) and validation set (Additional file 15: Table S6).

### 3.3 Development and assessment of individualized radiotranscriptomics signature‐based predictive nomograms

**Figure 5:**
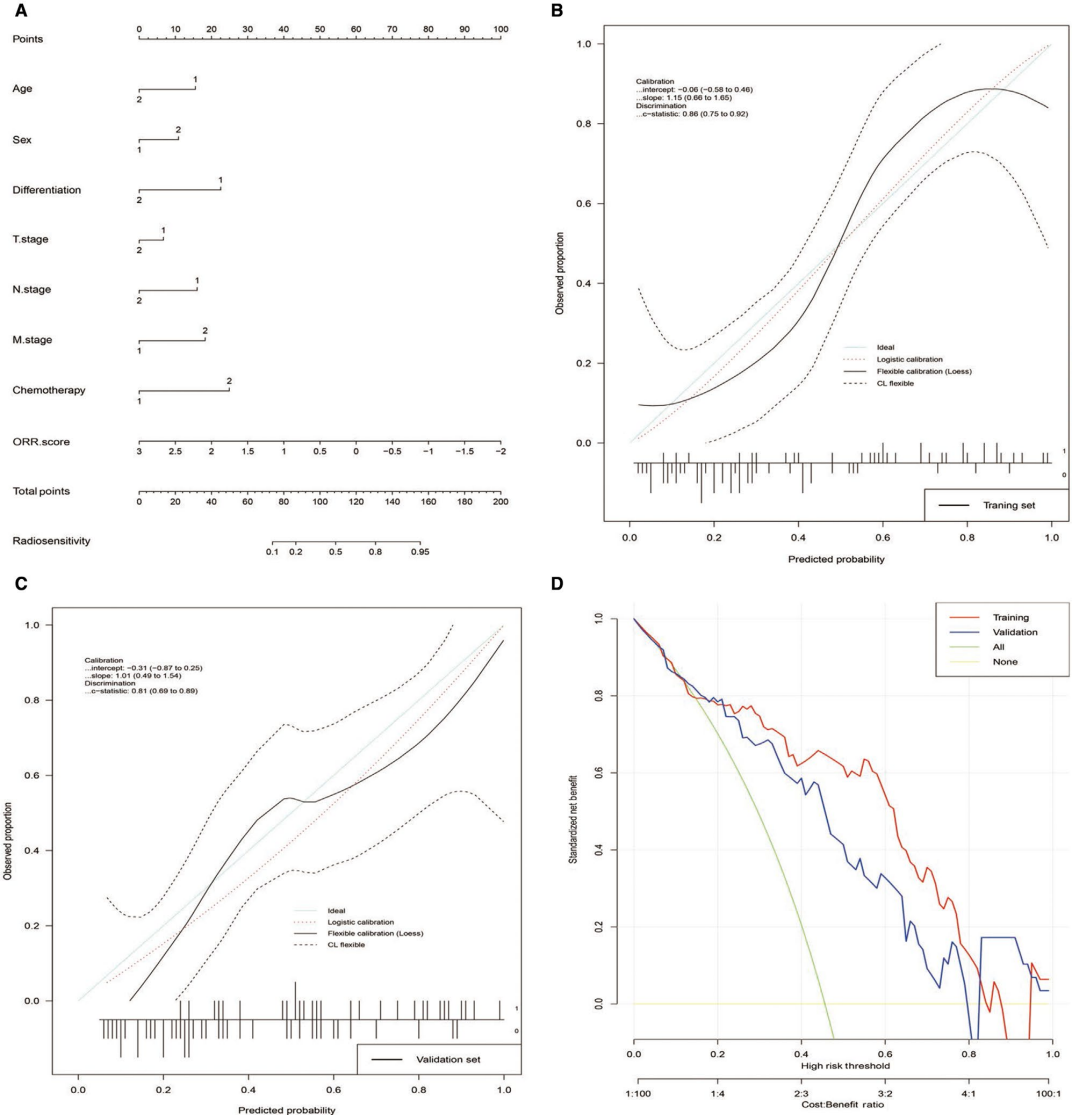
Radiotranscriptomics based nomogram development, assessment, and clinical use for objective response rate (ORR). A, The nomogram was developed in the training set incorporating age (1: ≤60, 2:> 60), sex (1: Women, 2: Men), differentiation (1: Well and Moderate, 2: Poor), T stage (1: T1 and T2, 2: T3 and T4), N stage (1: N0 and N1,2: N2 and N3), M stage(1:M0, 2:M1), chemotherapy (1: Nonplatinum drugs, 2: Platinum drugs only or both nonplatinum and platinum drugs), and ORR score. C,D, Calibration curves of the nomogram in both training and validation sets. D, Decision curve analysis for the nomogram model in both training and validation sets

Then, we tried to construct nomograms to predict the ORR, OS, and PFS of patients with NSCLC who received radiotherapy. After substantial attempts, only the ORR nomogram performed well, which included age, sex, differentiation, T stage, N stage, M stage, chemotherapy, and ORR score (Figure 5A). The C‐index values for the ORR nomogram were 0.86 (95% CI, 0.75 to 0.92) in the training set and 0.81 (95% CI, 0.69 to 0.89) in the validation set. The calibration curves showed good agreement between the nomogram‐evaluated probabilities and the actual probabilities in both the training set (Figure 5B) and validation set (Figure 5C). The calibration‐in‐the‐large was −0.06 (95% CI, −0.06 to 0.46) in the training set and −0.31 (95% CI, −0.87 to 0.25) in the validation set. The calibration slope was 1.15 (95% CI, 0.66 to 1.65) in the training set and 1.01 (95% CI, 0.49 to 1.54) in the validation set. Finally, we used DCA to evaluate the clinical use of the nomogram. DCA showed that the nomogram added more net benefit than both the “treat‐all‐patients” or the “treat‐more” strategies in most of the 0 to 1 threshold probabilities (Figure 5D) in both the training set and validation set. The nomogram including the radiotranscriptomics signature performed better than those that included clinical factors only (Additional file 16: Table S7).

### 3.4 Statistical relationship exploration between serum miRNA levels and CT texture features

Finally, we used LASSO to select the CT texture features related to serum miRNA levels and used a logistic regression model for signature generation in the training set. The results showed that the miR‐1290‐related image features formula was miR‐1290 Score = 0.1775‐1.1625 × GLCM_Dissimilarity.2; the miR‐25‐5p‐related image features formula was miR‐25‐5p Score = 0.2603685 − 0.7268367 × GLCM_Dissimilarity.2‐0.0159088 × GLZLM_SZLGE − 0.0007964 × GLZLM_LZLGE; the miR‐92a‐1‐5p‐related image features formula was miR‐92a‐1‐5p Score = −0.2085 + 1.34 × GLCM_Dissimilarity.2 − 0.00004615 × GLRLM_LRHGE. No miR‐2861‐related image features were found in our cohort. Next, we evaluated the performance of the image‐related features in discriminating differential miRNA levels by assessing the ROC curves in both the training and validation sets (Additional file 7, 8, and 9: Figures [Link], [Link], [Link]).

## 4 DISCUSSION

Our study provides evidence of the potential of radiotranscriptomic signatures to predict radiotherapeutic effects. We first tried to identify radiosensitivity‐related miRNAs. Then, we combined miRNA levels and CT texture features to generate radiotranscriptomics signatures to predict the objective response rate (ORR), overall survival (OS) and progression‐free survival (PFS) in patients with NSCLC. After calibrating the efficiency of the signatures by univariate and multivariate analyses, we constructed radiotranscriptomic signature‐based nomograms. Finally, we investigated the relationship between the CT texture features and serum miRNA levels.

miRNAs are naturally occurring small noncoding RNA molecules that are involved in almost all biological processes by regulating gene expression posttranscriptionally, and they play important roles in cellular homeostasis.^16^ Since miRNAs are relatively steady and widely exist in both intracellular and extracellular fluid, they are possible biomarkers of disease.^17^ In particular, because of the noninvasive nature of collecting whole blood or blood components such as serum and plasma, miRNAs in serum and plasma are ideal candidates.^18^ Compared to the significant efforts that have been devoted to the development of miRNAs as diagnostic biomarkers, efforts focused on their function as predictors for therapeutic effects started late but are meaningful.^19^ In this study, we first took advantage of established radioresistant (RR) cell lines to identify differentially expressed miRNAs between RR cells and their parental cell lines, which could be regarded as potential biomarkers for predicting radiosensitivity. After verification in vitro, a four‐miRNA signature was selected for clinical study. We prospectively gathered an NSCLC cohort, collected patients’ information, including the levels of the four miRNAs in serum as well as their clinical characteristics and image features, to integrate them and establish predictive models for radiotherapy effects. We evaluated the short‐term effect by radiosensitivity according to RECIST 1.1 and the long‐term effect by OS or PFS.

Noninvasive methods and tumor radiological imaging, such as CT and magnetic resonance imaging (MRI), are increasingly being used to correlate tumorigenesis, progression, and treatment response, using indicators such as lymph node metastasis, objective response rate, and survival.^20^, ^21^, ^22^ This led to the development of “radiomics.” In this work, we obtained CT images from our NSCLC cohort and employed CT texture features. However, the resulting interpretation of the radiomics signature was unsatisfactory. Based on previous experience, radiomics in combination with genomics seemed likely to be a better predictive tool,^23^ but there have been few attempts to combine the study of miRNAs with radiomics. To make the best use of our preliminary experimental results, we added the miRNA levels into the model building process, resulting in what we termed a radiotranscriptomic signature. This addition improved the predictive efficiency of the model.

Multiple clinical factors contribute to the treatment results in addition to molecular and radiological factors. Compared to traditional TNM stage prediction, nomograms have the advantages of integrating other clinical or statistically significant predictive factors and provide easily understandable interfaces for personalized medicine.^24^ Therefore, after defining the radiotranscriptomics signature, we tried to integrate them with other clinical factors and construct a nomogram to assist clinical decision‐making as has been done for many other cancer types.^25^, ^26^ Following nomogram construction, we evaluated the performance in both the training and validation sets by calibration curves and clinical utility by DCA. Hereto, we have completed the phased research task.

This research has some limitations as well as some promising future research directions. First, the sample size was inadequate, which may have led to selection bias and affected model building. Moreover, patients should be enrolled from multiple centers for more credible and generalizable results. Second, the current radiological analysis did not take into account intratumoral heterogeneity; this is a widespread challenge that requires a more sophisticated image‐processing method.^27^ Third, although our work preliminarily explored the correlation between CT texture features and miRNA levels, the underlying mechanism and causal relationship deserve further in‐depth study. Finally, the nomograms assumed that the data were static in time, and our observational indicators, either serum miRNA levels or CT texture features, occurred at one timepoint only. Thus, the results became less accurate with time. Further research should use dynamic detection to overcome this problem.

## 5 CONCLUSIONS

In conclusion, we identified the possibility of a combination of serum miRNA levels and image features as a potential future research direction for radiogenomics. In the present work, we constructed and validated pretreatment radiotranscriptomics signatures to predict ORR, OS, and PFS in NSCLC patents who received radiotherapy. Furthermore, we developed a well‐performing radiotranscriptomics signature‐based nomogram for ORR prediction. This nomogram provides a user‐friendly digital interface for clinical use.

## CONFLICT OF INTEREST

There is no conflict of interest disclosed in this study.

## AUTHOR CONTRIBUTIONS

Liyuan Fan contributed to conceptualization, methodology, data analyses, writing original draft, writing review and editing, and project administration. Qiang Cao contributed to methodology, data analyses, and validation. Xiuping Ding contributed to investigation, resources, data analyses, and project administration. Dongni Gao and Qiwei Yang contributed to writing review and editing. Baosheng Li contributed to conceptualization, supervision, and funding acquisition.

## Supporting information

Fig S1

Fig S2

Fig S3

Fig S4

Fig S5

Fig S6

Fig S7

Fig S8

Fig S9

Table S1

Table S2

Table S3

Table S4

Table S5

Table S6

Table S7

## Data Availability

The data analyzed during this study are available from corresponding author on reasonable request.
