# Corrigendum

**DOI:** 10.1002/cam4.3585

**Published:** 2020-12-06

In the article by Fan et al,^1^ the authors have noticed an error in Figure 2 and Table S1.

**Figure 2:**
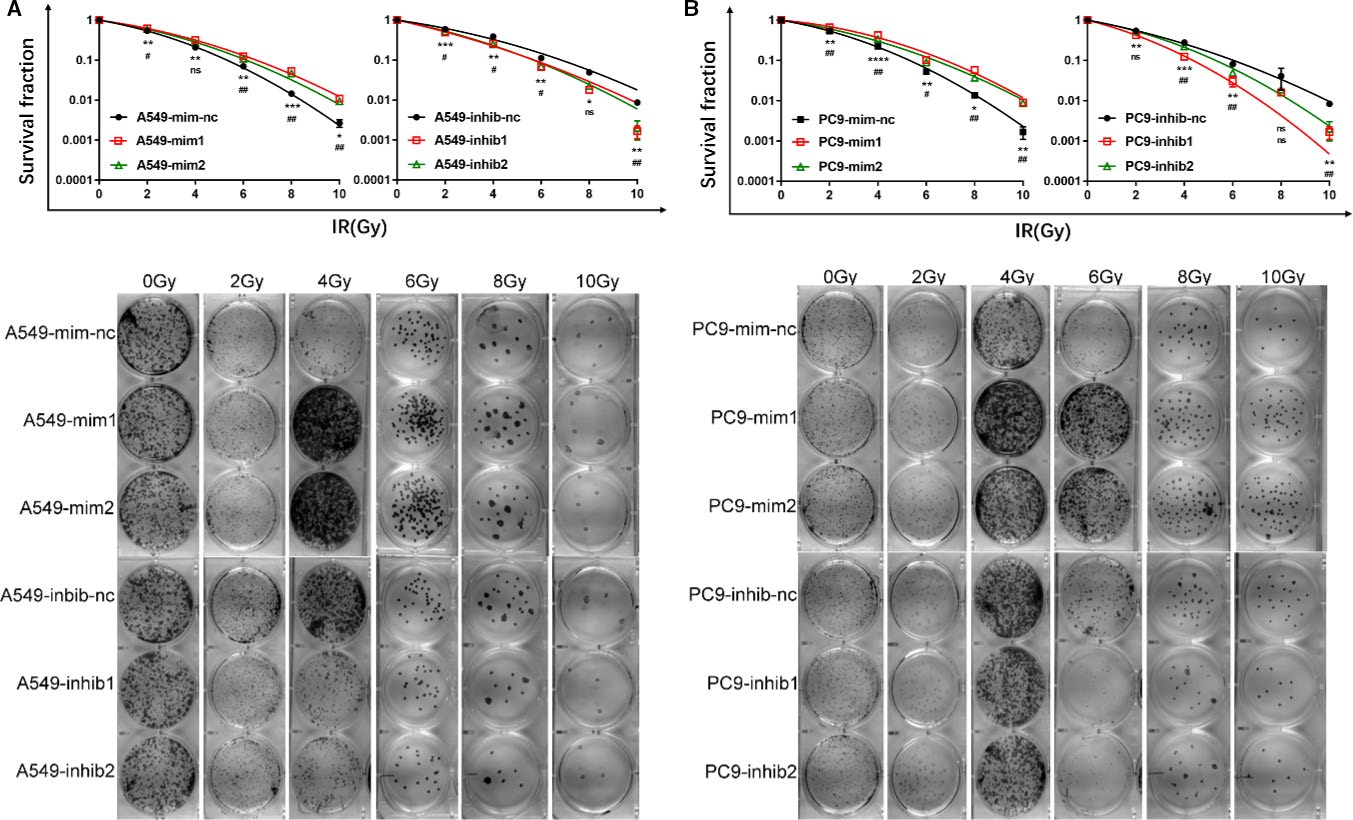
MiRNAs increase or decrease NSCLC cells’ radiosensitivity in vitro. n = 3 per group. mim1 = miR‐1290 mimics, mim2 = miR‐2861 mimics, inhib1 = miR‐25‐5p inhibitor, inhib2 = miR‐92a‐1‐5p inhibitor

The authors have mistakenly put the results of the pre‐experiment in Figure 2, and some information were displaced due to the original image coding error. The correct version of Figure 2 is displayed below:

While in Table S1, the same sequences are listed for both miR‐92a‐1‐5p‐RT and miR‐25‐5p‐R (“Primers for qRT‐PCR”). The correct sequences for miR‐25‐5p‐R can be found in the corrected version of Table S1.

The authors apologize for these errors, and assure the readers that the corrections will not alter the conclusion of the results.
