# Supplementary figures and images for: Radiotranscriptomics signature‐based predictive nomograms for radiotherapy response in patients with nonsmall cell lung cancer: Combination and association of CT features and serum miRNAs levels
Source: Cancer Med. 2020 May 27;9(14):5065–74. doi: 10.1002/cam4.3115 (PMC7367624; doi:10.1002/cam4.3115)

Percentage change from baseline  $\Delta$

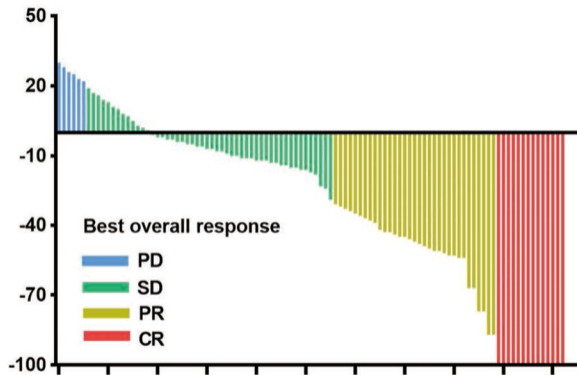

Percentage change from baseline  $\Delta$

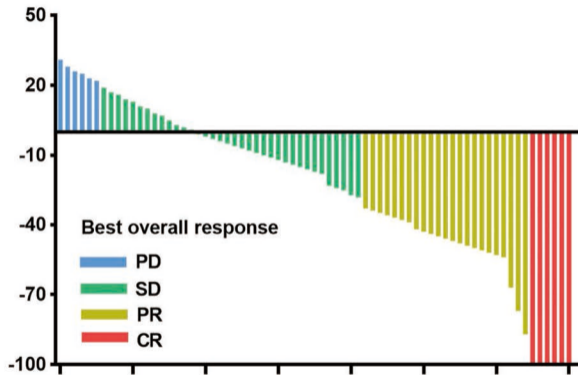

Supplement: Supplementary file 1 — Fig S1 [file CAM4-9-5065-s001.pdf]

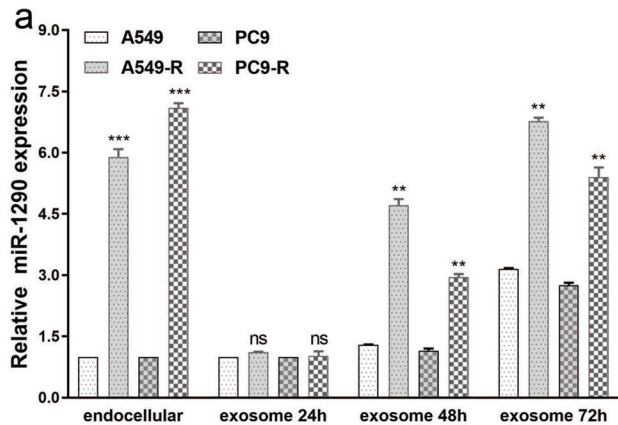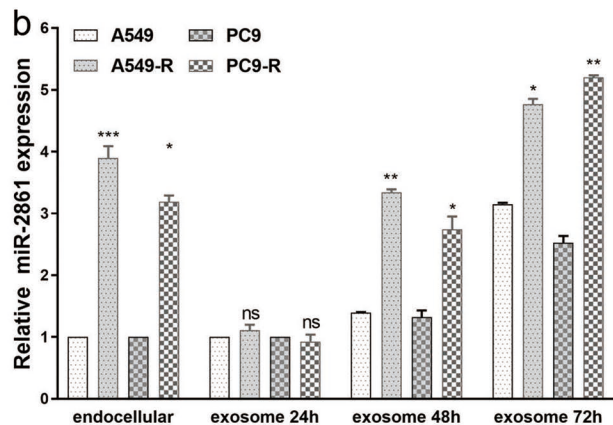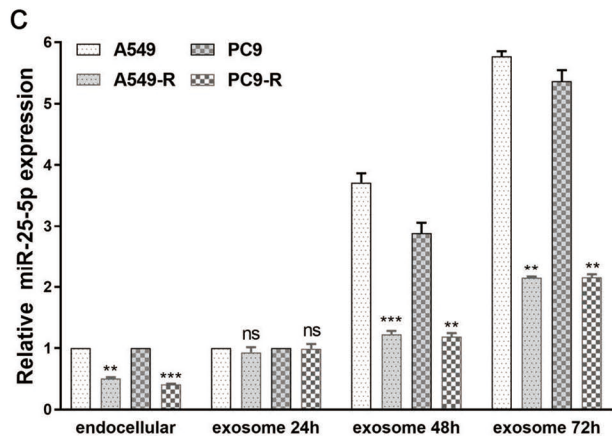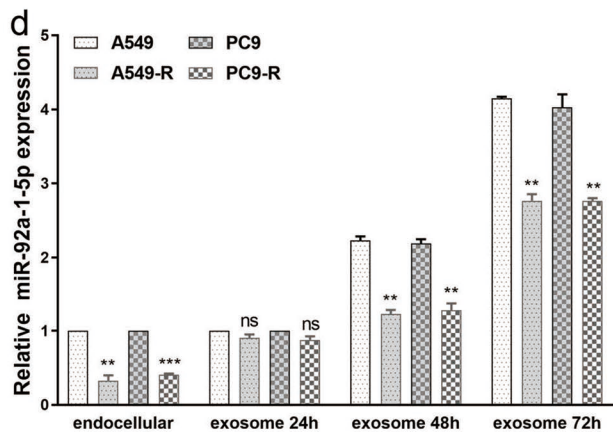

Supplement: Supplementary file 2 — Fig S2 [file CAM4-9-5065-s002.pdf]

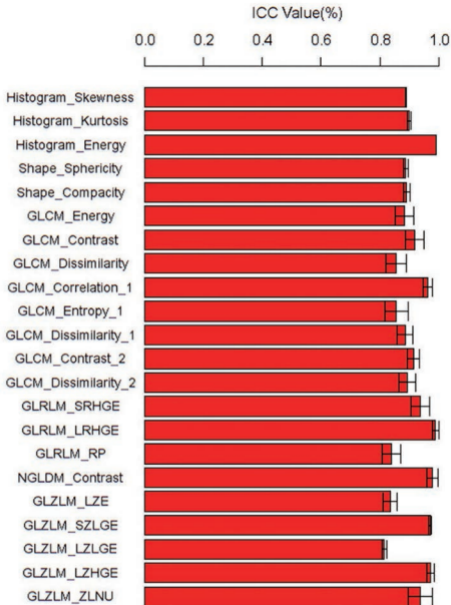

Supplement: Supplementary file 3 — Fig S3 [file CAM4-9-5065-s003.pdf]

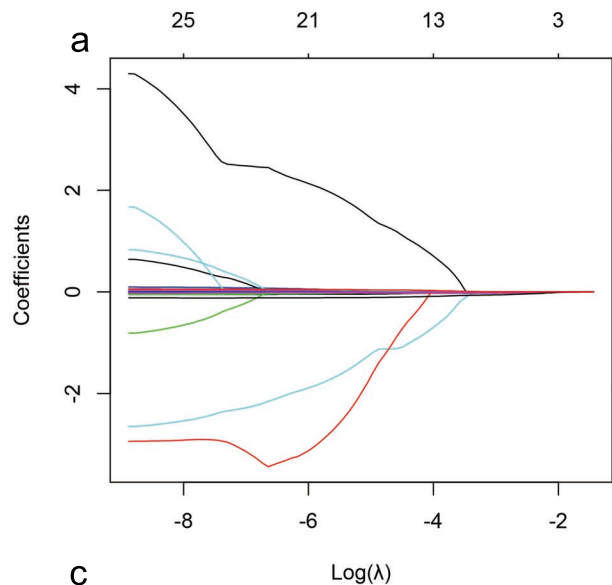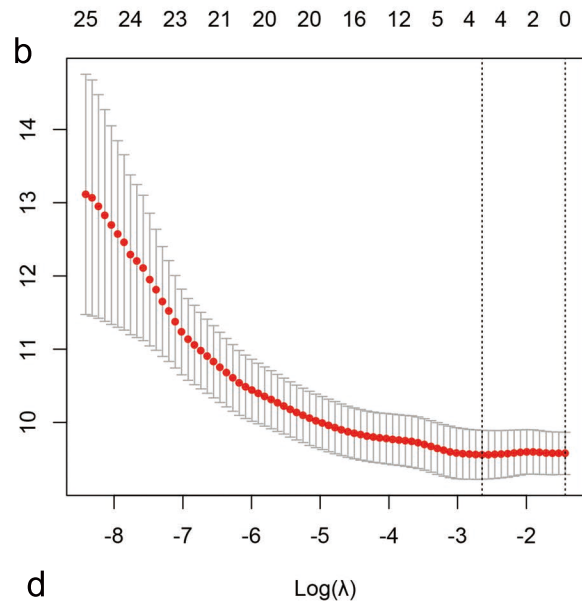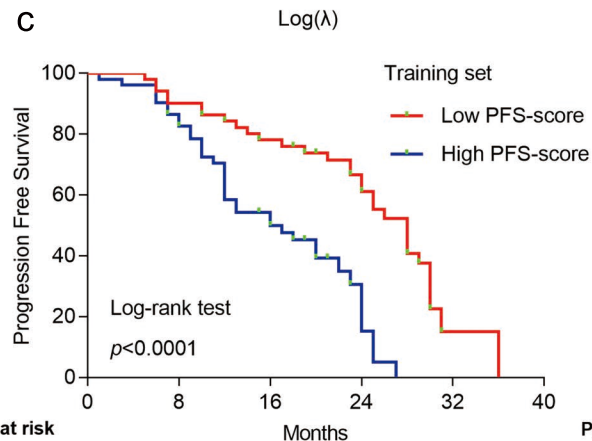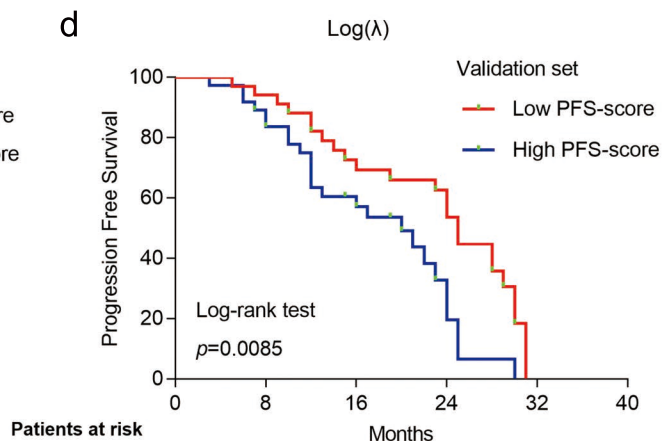

**Patients at risk**

|                | 0  | 8  | 16 | 24 | 32 | 40 |
|----------------|----|----|----|----|----|----|
| Low PFS-score  | 51 | 46 | 37 | 21 | 1  | 0  |
| High PFS-score | 52 | 41 | 21 | 3  | 0  | 0  |

**Patients at risk**

|                | 0  | 8  | 16 | 24 | 32 | 40 |
|----------------|----|----|----|----|----|----|
| Low PFS-score  | 34 | 32 | 21 | 12 | 0  | 0  |
| High PFS-score | 37 | 29 | 16 | 3  | 0  | 0  |

Supplement: Supplementary file 4 — Fig S4 [file CAM4-9-5065-s004.pdf]

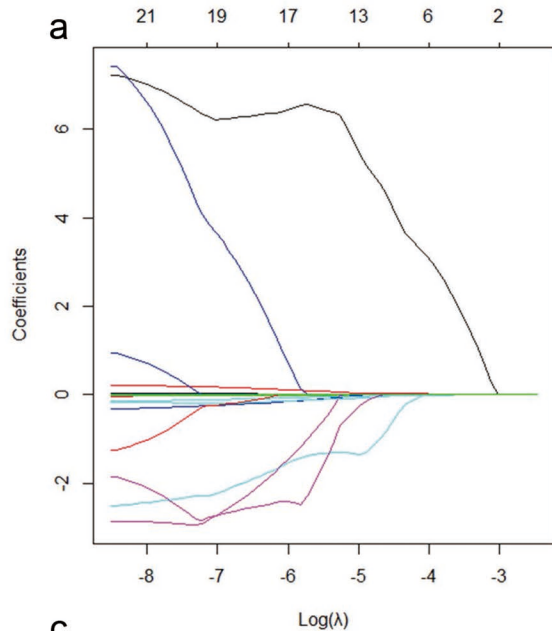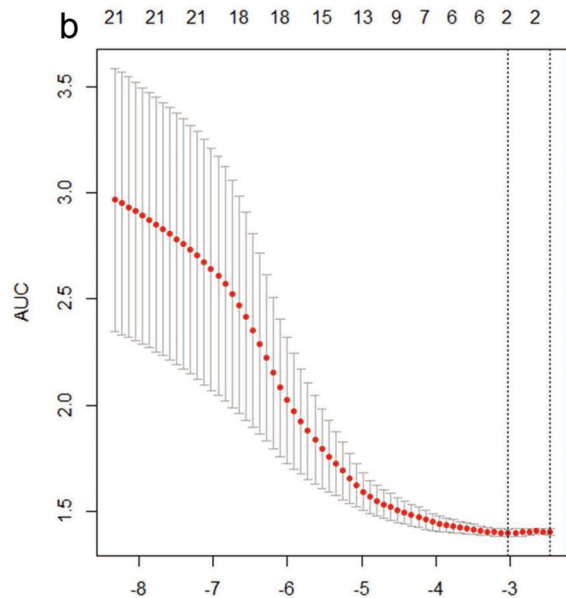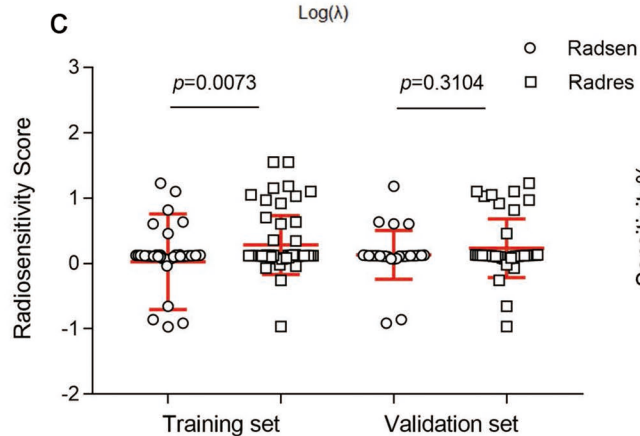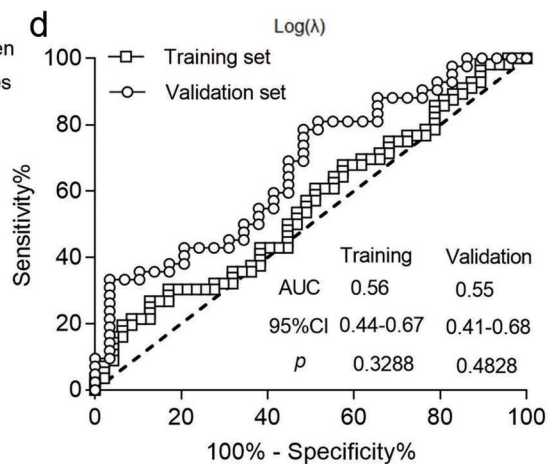

Supplement: Supplementary file 5 — Fig S5 [file CAM4-9-5065-s005.pdf]

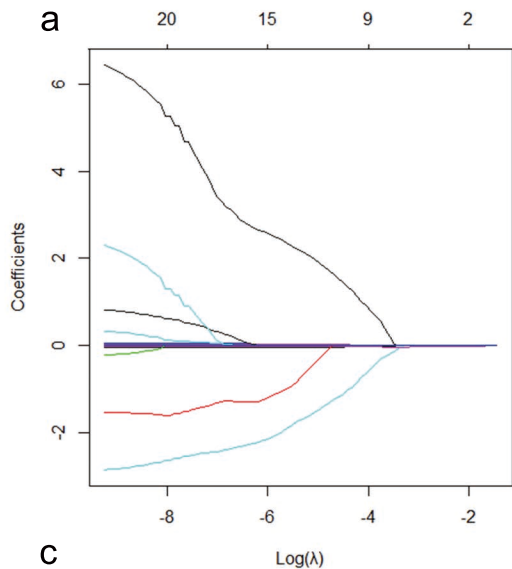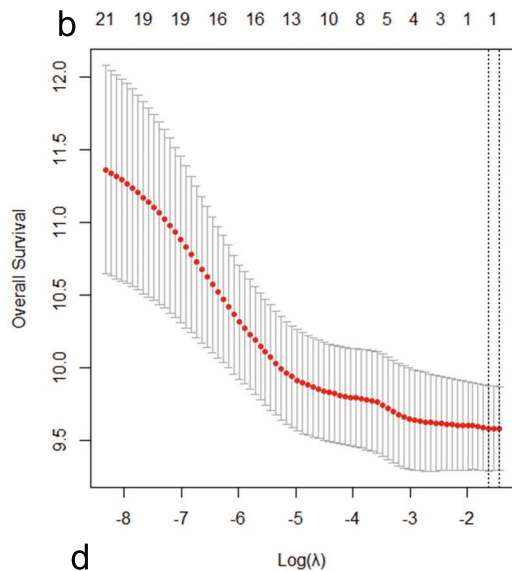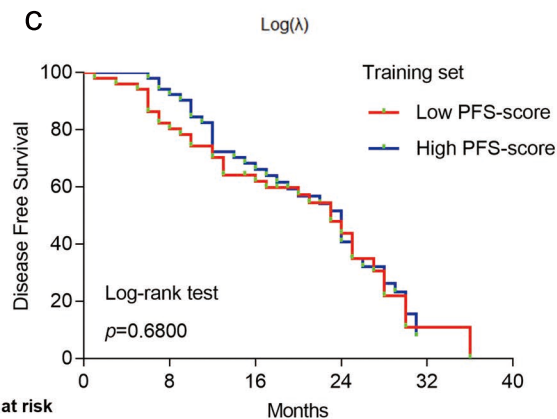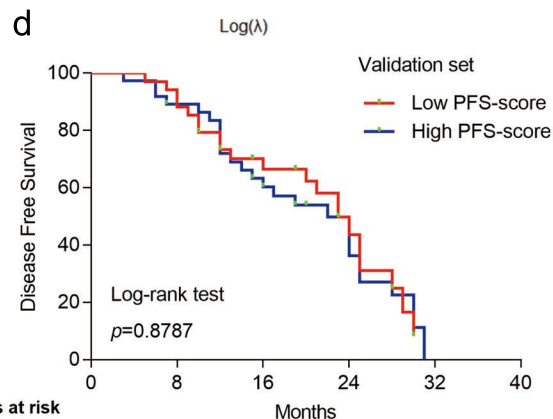

Supplement: Supplementary file 6 — Fig S6 [file CAM4-9-5065-s006.pdf]

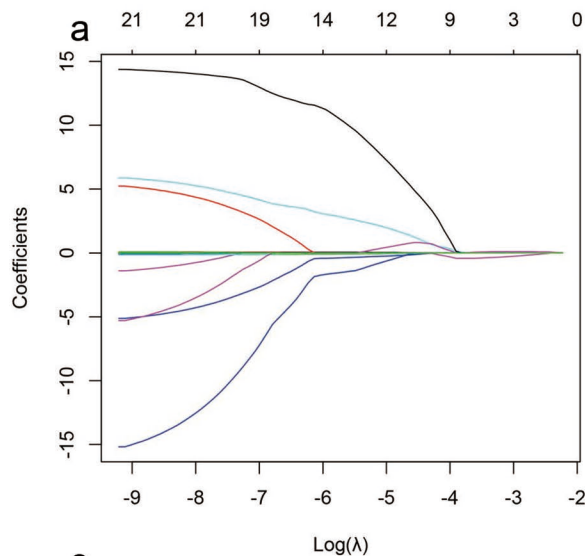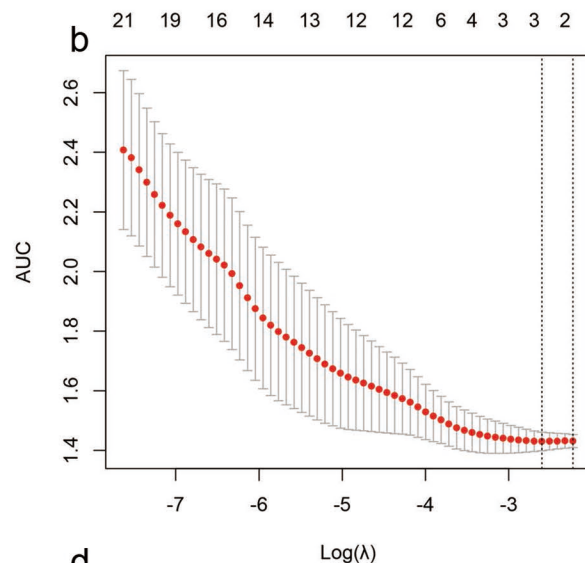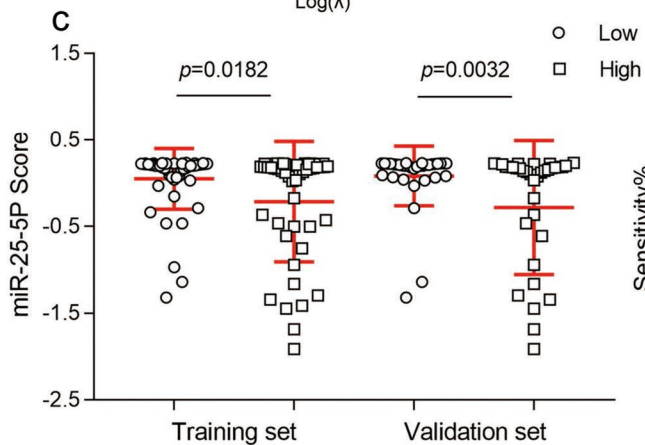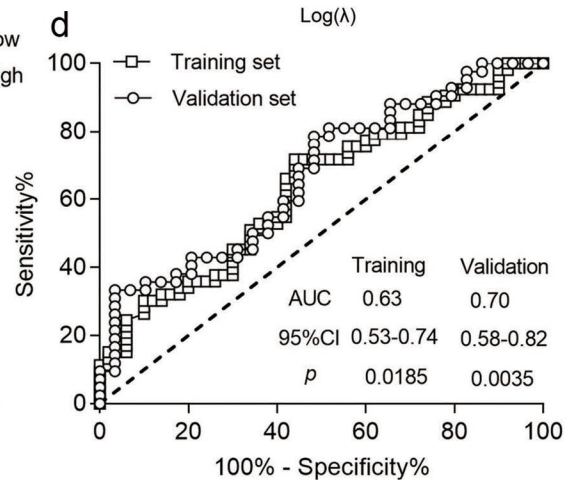

Supplement: Supplementary file 7 — Fig S7 [file CAM4-9-5065-s007.pdf]

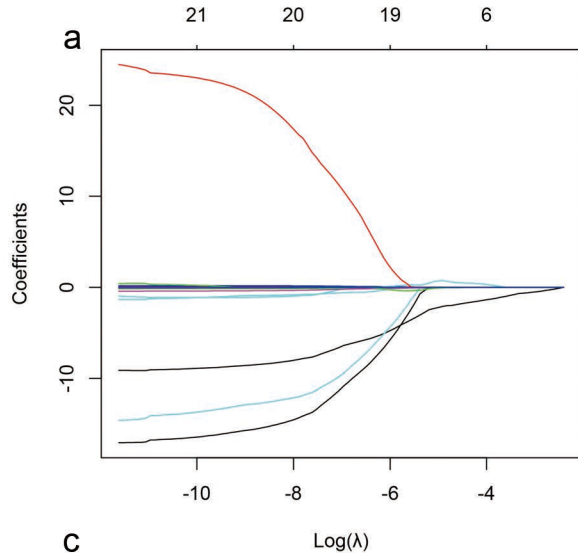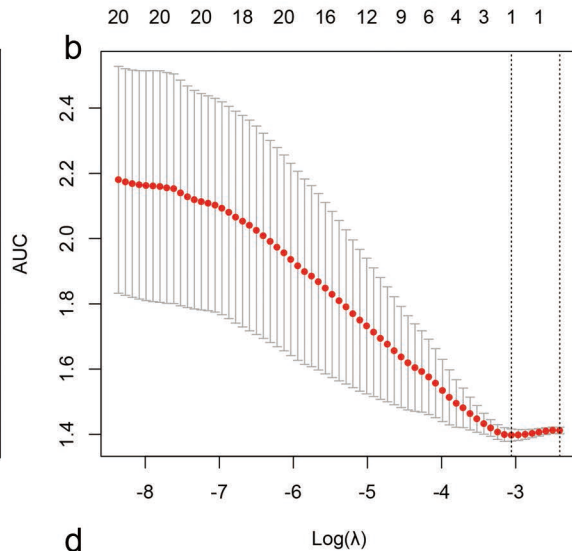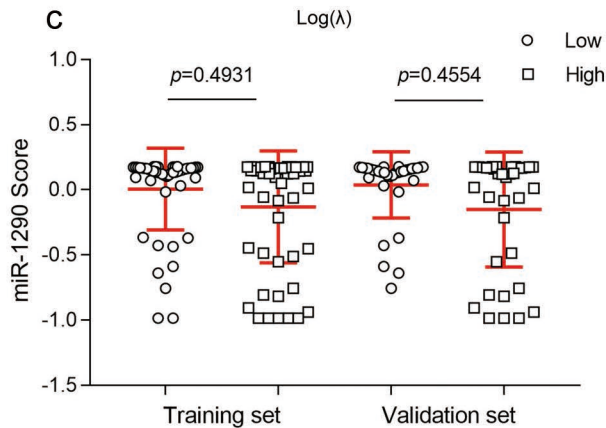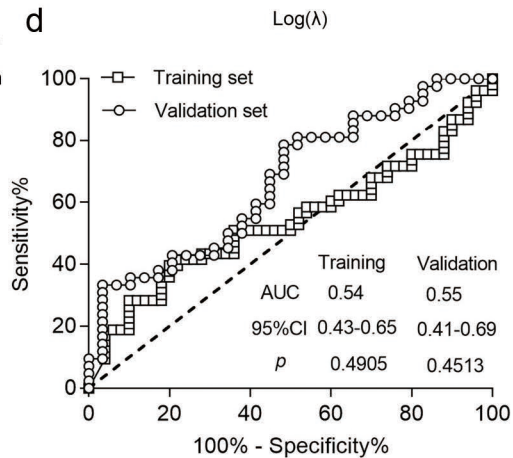

Supplement: Supplementary file 8 — Fig S8 [file CAM4-9-5065-s008.pdf]

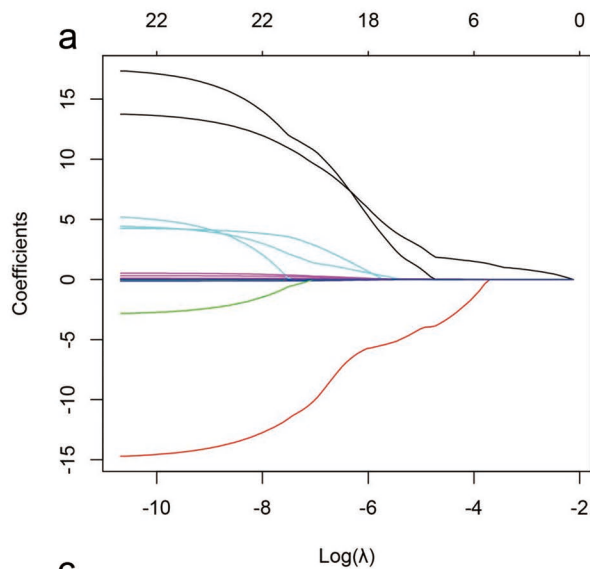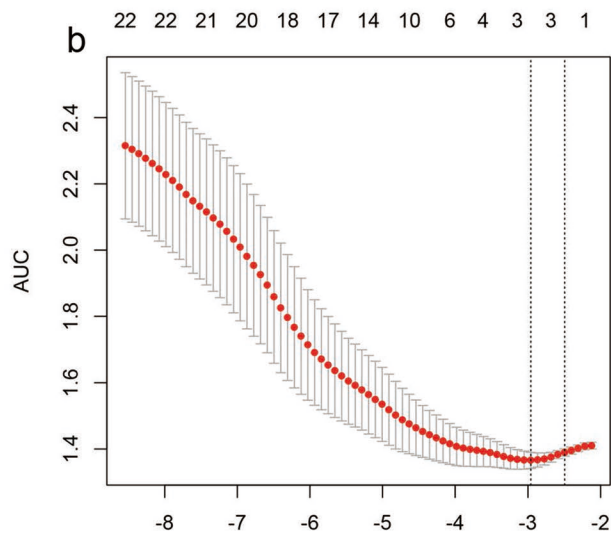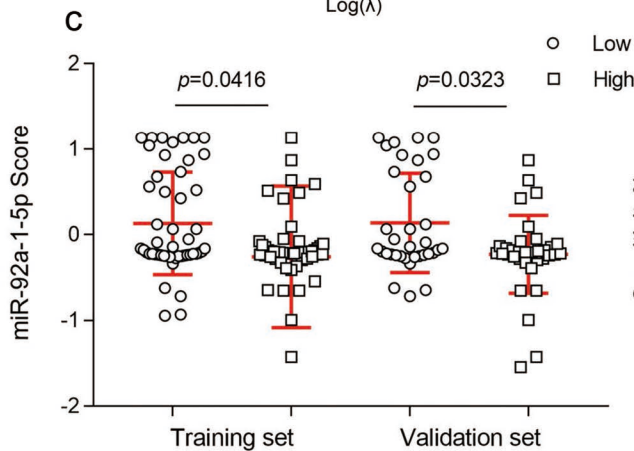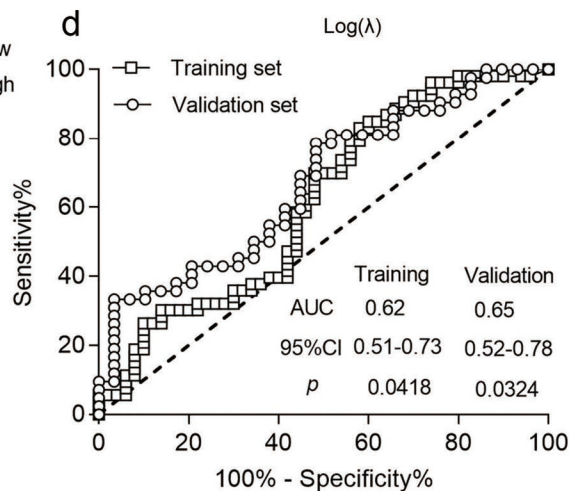

Supplement: Supplementary file 9 — Fig S9 [file CAM4-9-5065-s009.pdf]
