# Supplementary material for: Radiotranscriptomics signature‐based predictive nomograms for radiotherapy response in patients with nonsmall cell lung cancer: Combination and association of CT features and serum miRNAs levels
Source: Cancer Med. 2020 May 27;9(14):5065–74. doi: 10.1002/cam4.3115 (PMC7367624; doi:10.1002/cam4.3115)
Supplement: Supplementary file 10 — Table S1 [file CAM4-9-5065-s010.docx]

**Table S1:** Primers used in this study

| **Name** | **Sequence (5’-3’)** |
| --- | --- |
| Primers for qRT-PCR  miRNA universal R  miR-1290-F  miR-1290-RT  miR-2861-F  miR-2861-RT  miR-92a-1-5p-F  miR-92a-1-5p -RT  miR-25-5p-F  miR-25-5p-R  U6-F  U6-R | GTGCAGGGTCCGAGGT  AGC GTG TGT CGT GGA GTC  GTCGTATCCAGTGCGTGTCGTGGAGTCGGCAATTGCACTGGATAC  ACACTCCAGCTGGGGGGGCCTGGCGGT  CTCAACTGGTGTCGTGGAGTCGGCAATTCAGTTGAGCCGCCCAC  ATAAGGTTGGGATCGGTTGCA  GTCGTATCCAGTGCAGGGTCCGAGGTATTCGCACTGGATACGAC  CGCCTCTTTGGTTATCTAGCTGT  GTCGTATCCAGTGCAGGGTCCGAGGTATTCGCACTGGATACGAC  CTCGCTTCGGCAGCACA  AACGCTTCACGAATTTGCGT |

F: forward, R: reverse, RT: reverse-transcription.
