# Supplementary material for: Radiotranscriptomics signature‐based predictive nomograms for radiotherapy response in patients with nonsmall cell lung cancer: Combination and association of CT features and serum miRNAs levels
Source: Cancer Med. 2020 May 27;9(14):5065–74. doi: 10.1002/cam4.3115 (PMC7367624; doi:10.1002/cam4.3115)
Supplement: Supplementary file 11 — Table S2 [file CAM4-9-5065-s011.docx]

**Table S2:** The extracted features in CT image.

| **Feature categories** | **Symbol/abbreviation** |
| --- | --- |
| Histogram | Skewness_H, Kurtosis_H, Entropy_H, Energy_H; |
| Shape | Sphericity (only for 3D ROI (nZ>1), Compacity only for 3D ROI (nZ>1), Texture parameters, Distance of neighbours, Number of Grey Levels, Intensity Resampling, Bounds range of value after discretization, Z Spatial Resampling, Y Spatial Resampling, X Spatial Resampling; |
| GLCM matrix | Homogeneity^1^, Energy^1^, Contrast^1^, Correlation^1^, Entropy^1^, Dissimilarity^1^, Homogeneity-1^2^, Energy-1^2^, Contrast-1^2^, Correlation-1^2^, Entropy-1^2^, Dissimilarity-1^2^, Homogeneity-2^3^, Energy-2^3^, Contrast-2^3^, Correlation-2^3^, Entropy-2^3^, Dissimilarity-2^3^; |
| GLRLM matrix | SRE, LRE, LGRE, HGRE, SRLGE, SRHGE, LRLGE, LRHGE, GLNU, RLNU, RP; |
| NGLDM matrix | Coarseness, Contrast, Busyness; |
| GLZLM matrix | SZE, LZE, LGZE, HGZE, SZLGE, SZHGE, LZLGE, LZHGE, GLNU, ZLNU, ZP; |

1. Distance of neighbors is 1cm;
2. Distance of neighbors is 2cm;
3. Distance of neighbors is 4cm.
