# Supplementary material for: Radiotranscriptomics signature‐based predictive nomograms for radiotherapy response in patients with nonsmall cell lung cancer: Combination and association of CT features and serum miRNAs levels
Source: Cancer Med. 2020 May 27;9(14):5065–74. doi: 10.1002/cam4.3115 (PMC7367624; doi:10.1002/cam4.3115)
Supplement: Supplementary file 12 — Table S3 [file CAM4-9-5065-s012.docx]

**Table S3:** Selected differential expressed miRNAs by microarray and validated by qRT-PCR.

| miRNA | q-value (%)^1^ | FC^2^ | Alignments | Clustered miRNAs within 10kb | Target Genes^2^ | Microarray^3^ | qRT-PCR^3^ |
| --- | --- | --- | --- | --- | --- | --- | --- |
| hsa-miR-2861 | 0 | 5.36 | chr9:130548250-130548268 (+) | hsa-mir-3960 | AL627309.1, ZNF488, SPTBN5 | Up | Up |
| hsa-miR-4298 | 0 | 0.32 | chr11:1880735-1880756 (-) | --- | GCSAM, KSR2, YWHAZ | Down | - |
| hsa-miR-1290 | 0 | 19.18 | chr1:19223572-19223590 (-) | --- | HIGD2A, WDR77, OGN | Up | Up |
| hsa-miR-92a-1-5p | 4.57 | 0.22 | chr13:92003578-92003600 (+) | hsa-mir-17, hsa-mir-18a, hsa-mir-19a, hsa-mir-19b-1, hsa-mir-20a, hsa-mir-92a-1 | LMLN, ATN1, CXCL9 | Down | Down |
| hsa-miR-25-5p | 4.57 | 0.21 | chr7:99691233-99691253 (-) | hsa-mir-25, hsa-mir-93, hsa-mir-106b | FLJ00104, KLK9, MS4A4E | Down | Down |

FC: fold change; 1. The false discovery rate (FDR) of judging the miRNAs as differential expressed ones. The more significant the difference, the smaller q-value (%). 2.The first three target genes predicted by Targetscan. 3.The results of comparing radio resistant cell lines to parent ones.
