# Supplementary material for: Radiotranscriptomics signature‐based predictive nomograms for radiotherapy response in patients with nonsmall cell lung cancer: Combination and association of CT features and serum miRNAs levels
Source: Cancer Med. 2020 May 27;9(14):5065–74. doi: 10.1002/cam4.3115 (PMC7367624; doi:10.1002/cam4.3115)
Supplement: Supplementary file 13 — Table S4 [file CAM4-9-5065-s013.docx]

**Table S4:** Radiosensitive parameters of respective cell lines

| **Parameter** | **D0** | **SF2** | **SER10** |
| --- | --- | --- | --- |
| A549-mim-nc | 1.24 | 0.22 | 1 |
| A549-mim1 | 1.45 | 0.25 | 0.80 |
| A549-mim2 | 1.52 | 0.16 | 0.85 |
| A549-inhib-nc | 2.81 | 0.51 | 1 |
| A549-inhib1 | 2.99 | 0.64 | 1.19 |
| A549-inhib2 | 3.61 | 0.54 | 1.20 |
| PC9-mim-nc | 2.74 | 0.54 | 1 |
| PC9-mim1 | 3.71 | 0.68 | 0.79 |
| PC9-mim2 | 3.30 | 0.59 | 0.84 |
| PC9-inhib-nc | 3.13 | 0.54 | 1 |
| PC9-inhib1 | 2.22 | 0.40 | 1.39 |
| PC9-inhib2 | 2.88 | 0.49 | 1.12 |

mim1: miR-1290 mimics, mim2: miR-2861 mimics, inhib1: miR-92a-1-5p, inhib2: miR-25-5p; D0: dose to reduce survival to 37% of its value，SF2: surviving fraction at 2 Gy, SER10: sensitizer enhancement ratio at 10% survival
