# Supplementary material for: Radiotranscriptomics signature‐based predictive nomograms for radiotherapy response in patients with nonsmall cell lung cancer: Combination and association of CT features and serum miRNAs levels
Source: Cancer Med. 2020 May 27;9(14):5065–74. doi: 10.1002/cam4.3115 (PMC7367624; doi:10.1002/cam4.3115)
Supplement: Supplementary file 14 — Table S5 [file CAM4-9-5065-s014.docx]

**Table S5:** Multivariate analysis of ORR, OS, and PFS in the training set

| Characteristics | Subgroups | ORR | | | OS | | | PFS | | |
| --- | --- | --- | --- | --- | --- | --- | --- | --- | --- | --- |
|  |  | OR | 95% CI | *p* | HR | 95%CI | *p* | HR | 95% CI | *p* |
| Age | ≤ 60 vs > 60 | 0.61 | 0.28 to 1.34 | 0.045 | 0.49 | 0.25 to 0.96 | 0.038 | 0.90 | 0.53 to 1.54 | 0.698 |
| Sex | Female vs Male | 0.88 | 0.40 to 1.96 | 0.260 | 1.22 | 0.63 to 2.37 | 0.559 | 0.67 | 0.37 to 1.18 | 0.164 |
| Pathology | AC vs SCC | 0.76 | 0.35 to 1.66 | 0.270 | 0.70 | 0.39 to 1.29 | 0.255 | 1.46 | 0.81 to 2.62 | 0.212 |
| Differentiation | (Well & Moderate) vs Poor | 0.45 | 0.20 to 0.99 | 0.005 | 0.73 | 0.41 to 1.30 | 0.288 | 0.80 | 0.47 to 1.35 | 0.396 |
| Stage | I~II vs III~ IV | 1.25 | 0.69 to 2.25 | 0.377 | 0.54 | 0.15 to 1.93 | 0.343 | 1.00 | 0.36 to 2.81 | 0.990 |
| T stage | (T1&T2) vs (T3&T4) | 0.53 | 0.24 to 1.16 | 0.565 | 0.55 | 0.29 to 1.03 | 0.063 | 1.46 | 0.82 to 2.61 | 0.19 |
| N stage | (N0&N1) vs (N2&N3) | 0.49 | 0.22 to 1.13 | 0.405 | 0.32 | 0.13 to 0.78 | 0.012 | 1.00 | 0.47 to 2.12 | 0.99 |
| M stage | M0 vs M1 | 2.86 | 1.16 to 3.04 | 0.087 | 0.45 | 0.09 to 2.22 | 0.330 | 0.65 | 0.16 to 1.68 | 0.552 |
| Chemotherapy | N vs (P&NP) | 2.81 | 1.23 to 3.42 | 0.002 | 0.85 | 0.48 to 1.53 | 0.593 | 0.99 | 0.59 to 1.67 | 0.969 |
| Score | Higher vs Lower | 2.94 | 1.69 to 4.26 | <0.001 | 2.90 | 1.93 to 4.34 | <0.001 | 3.58 | 1.83 to 4.99 | 0.001 |
