# Supplementary material for: Radiotranscriptomics signature‐based predictive nomograms for radiotherapy response in patients with nonsmall cell lung cancer: Combination and association of CT features and serum miRNAs levels
Source: Cancer Med. 2020 May 27;9(14):5065–74. doi: 10.1002/cam4.3115 (PMC7367624; doi:10.1002/cam4.3115)
Supplement: Supplementary file 15 — Table S6 [file CAM4-9-5065-s015.docx]

**Table S6:** Multivariate analysis of ORR, OS, and PFS in the validation set

| Characteristics | Subgroups | ORR | | | OS | | | PFS | | |
| --- | --- | --- | --- | --- | --- | --- | --- | --- | --- | --- |
|  |  | OR | 95% CI | *p* | HR | 95% CI | *p* | HR | 95% CI | *p* |
| Age | ≤ 60 vs > 60 | 0.38 | 0.14 to 1.00 | 0.075 | 0.51 | 0.25 to 1.09 | 0.085 | 1.34 | 0.65 to 1.75 | 0.430 |
| Sex | Female vs Male | 0.76 | 0.29 to 1.98 | 0.839 | 1.04 | 0.46 to 2.33 | 0.931 | 0.93 | 0.46 to 1.91 | 0.859 |
| Pathology | AC vs SCC | 1.13 | 0.44 to 2.92 | 0.102 | 1.09 | 0.52 to 2.27 | 0.827 | 1.81 | 0.91 to 1.59 | 0.087 |
| Differentiation | (Well & Moderate) vs Poor | 0.27 | 0.10 to 0.74 | 0.080 | 0.56 | 0.28 to 1.14 | 0.111 | 0.69 | 0.36 to 1.32 | 0.264 |
| Stage | I~II vs III~ IV | 0.83 | 0.40 to 1.69 | 0.513 | 0.77 | 0.15 to 3.95 | 0.755 | 0.62 | 0.47 to 1.35 | 0.463 |
| T stage | (T1&T2) vs (T3&T4) | 0.43 | 0.17 to 1.14 | 0.332 | 0.42 | 0.18 to 0.99 | 0.046 | 0.82 | 0.38 to 1.81 | 0.630 |
| N stage | (N0&N1) vs (N2&N3) | 0.25 | 0.09 to 0.72 | 0.139 | 0.96 | 0.37 to 2.51 | 0.931 | 0.68 | 0.26 to 1.78 | 0.435 |
| M stage | M1 vs M0 | 1.93 | 0.67 to 5.59 | 0.196 | 0.69 | 0.09 to 1.44 | 0.728 | 0.46 | 0.28 to 1.46 | 0.367 |
| Chemotherapy | N vs (P&NP) | 0.76 | 0.29 to 1.98 | 0.815 | 0.74 | 0.35 to 1.56 | 0.430 | 1.12 | 0.56 to 1.23 | 0.754 |
| Score | Higher vs Lower | 2.94 | 1.45 to 3.88 | 0.026 | 2.14 | 1.26 to 3.64 | 0.004 | 2.64 | 1.19 to 3.81 | 0.016 |
