# Supplementary material for: Radiotranscriptomics signature‐based predictive nomograms for radiotherapy response in patients with nonsmall cell lung cancer: Combination and association of CT features and serum miRNAs levels
Source: Cancer Med. 2020 May 27;9(14):5065–74. doi: 10.1002/cam4.3115 (PMC7367624; doi:10.1002/cam4.3115)
Supplement: Supplementary file 16 — Table S7 [file CAM4-9-5065-s016.docx]

**Table S7:** Performance of ORR nomograms with or without radiotranscriptomics signature

| Performance measure | With radiotranscriptomics signature | | Without radiotranscriptomics signature | |
| --- | --- | --- | --- | --- |
|  | Training Set | Validation Set | Training Set | Validation Set |
| C-index | 0.86 | 0.81 | 0.77 | 0.72 |
| Calibration-in-the-large | -0.06 | -0.31 | -0.08 | -0.22 |
| Calibration Slope | 1.15 | 1.01 | 1.09 | 0.98 |
| DCA^*^ | 0.354 | 0.32 | 0.306 | 0.282 |

*Net Benefit at threshold 20%
